# Editorial: Optimizing probiotic applications in agriculture: Exploring the role of growth and health promoter's microorganisms in plants and livestock animals

**DOI:** 10.3389/fmicb.2022.993075

**Published:** 2022-08-09

**Authors:** Alice Checcucci, Diana Luise, Francesco Pini

**Affiliations:** ^1^Department of Agricultural and Food Science, University of Bologna, Bologna, Italy; ^2^Department of Biology, University of Bari-Aldo Moro, Bari, Italy

**Keywords:** probiotic, plant growth promoting microorganisms, livestock, sustainability, agriculture

At the current state, the agricultural and livestock production would not be able to sustain the exponential increase of global population expected for the next 50 years. Furthermore, the change in dietary habits in favor of a higher meat consumption, will increase the food demand in the coming decades (Salter, 2017).

Intensive agricultural farming also involves the use of substances causing agrochemical pollution such as chemical fertilizers, pesticides, and plant growth regulators, to control damages that may arise from environmental conditions or biotic stresses toward crops. Furthermore, the large use of antibiotics to sustain livestock production would lead to the emerging of antibiotic resistant strains and to a decrease of the overall microbial biodiversity (Aidara-Kane et al., 2018).

The need for a sustainable increase of agricultural productivity has then become one of the most important challenges of the last 25 years. The slowing of environmental degradation (due to deforestation practices) and soil depletion (due to the overuse of agricultural land) are among the primary goals for forward-looking and non-impacting agricultural practices (Borrelli et al., 2017).

Probiotics are defined as beneficial microbes, conferring health benefits to the host whether it is a plant or an animal. In this sense, in the last years the exploitation of probiotic microorganisms for the plant wellness and growth has largely caught on, so much so that nowadays, bioinoculants based on plant growth promoting (PGP) microorganisms are fundamental in green agriculture (Maitra et al., 2022). PGP microorganisms are part of a complex microbial community which naturally colonize plants as endophytes diffusing in internal tissues and roots and as rhizobacteria diffusing in the rhizosphere and contributing to their biotic (such as pathogens) and abiotic stress tolerance like high salinity concentrations (Bellabarba et al., 2019).

PGP traits and abilities range over nitrogen fixation, phosphorus solubilization, 1-aminocyclopropane-1-carboxylate (ACC) deaminase, indole acetic acid (IAA) and siderophores production, biocontrol agents against pathogens production. These are the characteristics mostly used to screen for PGP rhizobacteria (PGPR); however other features may be useful for their identification. Shi et al. showed that the capability to use metabolites commonly found in root exudates could be an alternative approach for the screening of potential PGPR, as rhizosphere and roots colonization is a fundamental prerequisite to exert their PGP role.

Among the PGP traits mostly screened, one of particular interest is the resistance to water deficiency, as this issue will become more and more prominent in the next few years because of climate change. Riva et al. tested different PGP bacterial strains as bioinoculants on tomato plants in normal and water deficit conditions, showing a significant effect of the strains tested to increase the number of productive plants in a short-term assay.

In the past years, PGP strains have been isolated from many different plants, in particular from plants of commercial interest. PGP bacteria may be highly different within the same plant species exhibiting different functional roles to increase plant productivity. Gushgary-Doyle et al. have characterized three N_2_ fixing switchgrass endophytes, at genomic and phenotypic level, highlighting the presence of multiple PGP features in each strain, from nutrient mobilization to plant hormone production.

However, also less common plant species could be a potential resource for novel PGP bacteria; Jain et al. explored the cultivable endophytic community of *Arnebia euchroma*, a plant typical of the cold Himalayan desert, isolating several microorganisms (bacteria and fungi) with different PGP capabilities that may help their host to withstand in cold environments.

The use of probiotic bacteria is not limited to plants but is now increasing its interest as a potential strategy to reduce the use of antibiotics and antimicrobials to increase performance and sustain the health of livestock animals.

As reviewed by Luise et al. the use of *Bacillus* strains as a probiotic strategy can have promising results in terms of growth performance and health; contributing in a reduced post-weaning diarrhea in piglets and the mortality in broilers. The correct definition of probiotic strains and doses would allow achieving the same performance and health parameters obtained using antimicrobials.

Furthermore, probiotics strains can be used together with specific prebiotics to obtain a synergic effect as proposed by Rodríguez-Sorrento et al. The authors observed that the supplementation of *B. longum* subsp. *infantis* combined with a mixture of inulin and fructooligosaccharides may have promising results against infections due to *Salmonella enterica* serovar Typhimurium and *Escherichia coli F4*, which are two of the most relevant pathogens for piglets. The symbiotic combination was able to influence the fermentation and the immune system activities in the gut of post-weaning piglets, depending on the pathogen infection.

Overall, considering the potential of probiotics for improving plant and animal health, a continuous development and research would be necessary to identify new bacterial strains or new combinations of probiotics. Studies for a deeper characterization of PGP microorganisms and their in-planta effects should be necessary to foresee the induced physiological effects in plant growth and development. Furthermore, account for the consequences of probiotics inoculation on pre-existing plant microbiota could allow the development of highly specific bioinoculants, selected on the basis of plant genome and its microbiome.

The research for their use in livestock animals should aim to offset the specific requirements during the different growing phases, as well as the specific sanitary conditions/pathogen infections. Furthermore, the continuous collaboration between scientific research, industry and in-field figures is encouraged to facilitate the development of a practical guide to lead to a transition into a more sustainable production of meat, based on a reduction of antimicrobials use.

## Author contributions

All authors listed have made a substantial, direct, and intellectual contribution to the work and approved it for publication.

## Conflict of interest

The authors declare that the research was conducted in the absence of any commercial or financial relationships that could be construed as a potential conflict of interest.

## Publisher's note

All claims expressed in this article are solely those of the authors and do not necessarily represent those of their affiliated organizations, or those of the publisher, the editors and the reviewers. Any product that may be evaluated in this article, or claim that may be made by its manufacturer, is not guaranteed or endorsed by the publisher.
